# An ultra scale‐down method to investigate monoclonal antibody processing during tangential flow filtration using ultrafiltration membranes

**DOI:** 10.1002/bit.26859

**Published:** 2019-01-04

**Authors:** Lara Fernandez‐Cerezo, Andrea C. M. E. Rayat, Alex Chatel, Jennifer M. Pollard, Gary J. Lye, Michael Hoare

**Affiliations:** ^1^ Department of Biochemical Engineering University College London London UK; ^2^ Downstream Process Development and Engineering Merck and Co., Inc Kenilworth New Jersey

**Keywords:** CFD, membrane, monoclonal antibody diafiltration, TFF, ultra scale‐down

## Abstract

The availability of material for experimental studies is a key constraint in the development of full‐scale bioprocesses. This is especially true for the later stages in a bioprocess sequence such as purification and formulation, where the product is at a relatively high concentration and traditional scale‐down models can require significant volumes. Using a combination of critical flow regime analysis, bioprocess modelling, and experimentation, ultra scale‐down (USD) methods can yield bioprocess information using only millilitre quantities before embarking on highly demanding full‐scale studies. In this study the performance of a pilot‐scale tangential flow filtration (TFF) system based on a membrane flat‐sheet cassette using pumped flow was predicted by devising an USD device comprising a stirred cell using a rotating disc. The USD device operates with just 2.1 cm^2^ of membrane area and, for example, just 1.7 mL of feed for diafiltration studies. The novel features of the design involve optimisation of the disc location and the membrane configuration to yield an approximately uniform shear rate. This is characterised using computational fluid dynamics for a defined layer above the membrane surface. A pilot‐scale TFF device operating at ~500‐fold larger feed volume and membrane area was characterised in terms of the shear rate derived from flow rate‐pressure drop relationships for the cassette. Good agreement was achieved between the USD and TFF devices for the flux and resistance values at equivalent average shear rates for a monoclonal antibody diafiltration stage.

## 1 INTRODUCTION

Membrane‐based operations are ubiquitous in industrial processes including biomanufacturing. They offer the potential of scalability and robustness without requiring additional chemicals or harsh conditions to operate (Cui & Muralidhara, 2010; Lutz, 2015). The use of membranes in bioprocessing, for example of therapeutic proteins, includes initial removal of particulates such as cells or cell debris, impurity clearance using viral and sterile filters, concentration and diafiltration for feed preparation to chromatography stages or for final formulation (Rathore & Shirke, 2011). Membrane structures may be designed to retain macromolecules, generally termed ultrafiltration, or to retain particulates, generally termed microfiltration. Membrane operations may operate in either tangential flow (cross‐flow) filtration (TFF) mode to reduce fouling of the membrane surface, or in normal flow (dead‐end) filtration (NFF) mode often for the removal of trace particulates.

Industrial modules can be operated in different configurations including batch, fed‐batch, single‐pass, and feed‐and‐bleed/continuous (Holzer, 2017). Continuous or feed‐and‐bleed operations are common at large‐scale but for many bioprocessing applications, batch operation is used with the option of an additional feed for diafiltration stages (Cheryan, 1998; van Reis & Zydney, 2007). This article will focus on the use of an ultrafiltration membrane for a diafiltration stage operating in TFF batch mode. Membrane geometries for TFF include hollow fibre, spiral wound, and flat‐sheet, the last being the form typically found in bioprocessing for therapeutics (Lutz, 2015). The flat sheets are commonly mounted into cassettes, these being compact rectangular units with smaller footprint consisting of multiple flat sheet membranes layered with flow channel spacers. These spacers often incorporate turbulence promoters to help reduce membrane fouling and to increase the bulk mass transfer at the membrane surface (Shrivastava, Kumar, & Cussler, 2008). This format allows reduced hold‐up volumes and improved permeate flux, hence decreasing the exposure of process material to shear stress during pumping and flow (Lutz, 2015). Mechanically agitated systems have been devised to increase shear over the membrane whereas still operating at low flow rates, for example, using an axial rotating cylinder (Holeschovsky & Cooney, 1991; Ji et al., 2016; Kroner & Nissinen, 1988) or a rotating disc (Ebrahimi, Schmitz, Kerker, Liebermann, & Czermak, 2013).

Scale down of membrane systems to bench scale poses significant challenges. It is necessary to maintain flow path length and similar wall and entrance effects to help mimic hydrodynamic shear characteristics, while also ensuring the pumping and piping flow effects remain the same. Rayat, Lye, and Micheletti (2014) used an equivalent hydraulic length to account for flow disruption effects in a channel with changes in flow direction to successfully mimic the pressure drops and shear rates present in full‐scale TFF systems thereby reducing the membrane area used to 10 cm^2^. Stirred‐well systems have been used, where the intention is to maintain a clear membrane surface, to study environmental effects on protein processing such as pH and salt concentration using membrane areas of 0.25 cm^2^ in multi‐well plates (Kazemi & Latulippe, 2014) or the effects of membrane pore size and composition on the specific transmission of proteins using 1.5 cm^2^ membrane discs (LaRue, Kazemi, & Latulippe, 2018).

Membrane separation processes are often scaled by maintaining the same membrane loadings and membrane configurations (i.e., path length) and varying the number of membrane channels (i.e., the number of membrane sheets for cassette formats; van Reis et al., 1997). Scale‐down systems have been developed with capacity ratios of 1:100 to 1:400 resulting in membrane area requirements of 10–50 cm^2^ (Brose, Dosmar, Cates, & Hutchison, 1996; Rayat et al., 2014). These systems, however, still require 100s of millilitres of feed material. Often they do not directly mimic larger scale operation because of differences in the pump hydrodynamic environment, the required number of passes, and the magnitudes of shear rate (Meireles, Aimar, & Sanchez, 1991).

Ultra scale‐down (USD) technologies use experimentation at the millilitre scale to help understand the impact of the large‐scale process environment. USD conditions may be defined by combining critical flow regime analysis and bioprocess modelling of large‐scale systems (Rayat, Chatel, Hoare, & Lye, 2016; Titchener‐Hooker, Dunnill, & Hoare, 2008). USD techniques often incorporate the ability to mimic the full‐scale shear environment and flow patterns to study the impact on the stability of biological materials (Biddlecombe et al., 2007; Reid et al., 2010).

USD methodologies are able to offer a wider understanding of the effect of individual parameters by decoupling dependencies, for example, shear stress in the entry zone of a continuous flow industrial‐scale centrifuge, sedimentation in the settling zone, and shear stress during sediment discharge (Boychyn et al., 2001, 2004; Chan et al., 2006). The use of USD techniques has been described for chromatography (Wenger, Dephillips, Price, & Bracewell, 2007; Willoughby, Martin, & Titchener‐Hooker, 2004), NFF (Lau et al., 2013; Reynolds et al., 2003), for membrane separations in normal‐flow mode (Jackson, Liddell, & Lye, 2006; Rayat, Micheletti, & Lye, 2010), in cross‐flow mode using pumped flow (Rayat et al., 2014), and in mechanically‐agitated mode using a rotating disc (Ma et al., 2009). This latter form of the USD device allows the flow over the membrane to be varied independently of the transmembrane flux or pressure. It has been used to study microfiltration for antibody fragment recovery from clarified *Escherichia coli* lysates (Ma et al., 2009) and human cell recovery (Masri, Lawrence, Wall, & Hoare, 2017). This article addresses the challenge of characterizing the shear rate over the membrane in such a way that it can be related to shear rate in full‐scale operations. An important precursor is the need to redesign the USD device so that all the membrane may be considered to be exposed to the same shear rate.

The objective of this study was to characterise the redesigned USD device and the relationship to the performance of a flat‐sheet membrane cassette in a pilot‐scale TFF system. The membrane performance, using flux as a comparative measure, was studied for a diafiltration operation of a monoclonal antibody (mAb) solution. This type of operation was selected to solely focus on the membrane performance over time, i.e., while the protein concentration remains unchanged. Ultra scale‐down studies of membrane performance were carried out over a range of shear rates encompassing those which are observed at full scale. A characteristic average shear rate was the scaling parameter used to compare performance at the two scales.

## 2 MATERIALS AND METHODS

### 2.1 Materials

A humanized monoclonal antibody (IgG), referred to as mAb‐1 (~150 kDa, pI 9.0) was provided by Merck & Co., Inc (Kenilworth, NJ). It was supplied as two frozen (− 80°C) samples (2 L aliquot at 12 g/L) stored in 10 mM Sodium Acetate buffer pH 5.5. The frozen samples were thawed overnight before preparation through diafiltration into 10 mM Tris Acetate buffer pH 5.4. The resulting solution at 12 g/L was used for pilot‐scale TFF and USD studies within 24 hr of thawing. This concentration was representative of a feed for final membrane bioprocessing stages.

Pilot‐scale data was obtained using a 0.11 m^2^ membrane cassette (C‐screen Pellicon 3, Ultracel PLC^TM^, composite regenerated cellulose, molecular weight cut‐off (MWCO) = 30 kDa, EMD Millipore, Bedford, MA). USD data was obtained using membrane filter discs (Ø = 25 mm, Ultracel PLC^TM^, composite regenerated cellulose, MWCO = 30 kDa, EMD Millipore). All chemicals used for buffer preparation were from Sigma‐Aldrich (St. Louis, MO) unless otherwise stated. Buffers were vacuum filtered before use (Steritop^TM^ vacuum bottle‐top filters, 0.22 µm pore size, EMD Millipore).

### 2.2 Equipment

The rheology of the feed and diafiltration buffer (10 mM Tris Acetate pH 5.4) was determined using a capillary viscometer (m‐VROC, RheoSense^©^, San Ramon, CA) as a function of time, shear rate and temperature.

A purpose‐designed TFF unit was used for pilot‐scale studies (PendoTECH TFF Process Control System^TM^, PendoTECH, Princeton, NJ) fitted with a membrane cassette and operated using a quaternary diaphragm pump (QuattroFlow^TM^ 150 S, Triangle Process Equipment, Wilson, NC). Pressure was measured by sensors (PREPS‐N‐025, PendoTECH) located in the feed and retentate lines. The permeate line was left open to atmosphere. The diafiltration tank (10 L) and the feed tank (1 L) were linked via a peristaltic pump (Masterflex^®^ L/S^TM^, Cole‐Parmer Instrument Company, Vernon Hills, IL). The required mean transmembrane pressure drop, ΔP_TMP_, was achieved through automatic adjustment of a pinch‐valve on the retentate line. All pilot‐scale TFF trials were performed at 20.5 ± 0.5°C (room temperature).

**Figure 1:**
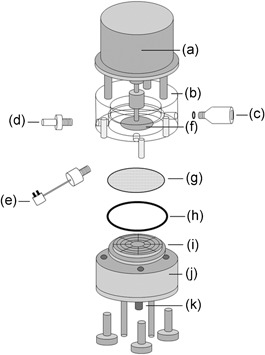
Simplified schematic representation of each individual component of the USD membrane system. There are ten main components: (a) motor; (b) perspex chamber with three ports; (c) pressure sensor; (d) feed syringe pump connector; (e) thermocouple; (f) rotating disc; (g) membrane; (h) seal; (i) filtration base to accommodate the membrane disc; (j) support frit providing a jacketed‐housing for cooling system; (k) permeate outlet port. Note that the drawing is not drawn to scale

The USD device was designed and fabricated in house (Rapid Design and Fabrication Facility, Department of Biochemical Engineering, UCL, UK) and is detailed in Figure 1. The device comprises a perspex chamber (*h* = 56 mm, *Ø* = 21 mm, 1.7 mL capacity) with a stainless steel base that includes a support frit to accommodate the membrane disc, a permeate outlet port, and a jacketed‐housing to provide a controlled temperature environment within the chamber by using a recirculating water bath (211‐131‐100, Fisher Scientific™, Loughborough, UK).

Stainless steel rotating discs of various designs (*Ø* = 15 mm) could be mounted at a specified distance (from 0.8 to 2.0 mm) above the membrane surface and driven at a specified speed up to 5,000 rpm using a top‐mounted stepper motor (D8‐MD81‐011, M‐1713‐1.5 S Schneider Electric Motion, Marlborough, MA). Selected areas of the membrane filter were blanked, reducing effective membrane area from 3.46 to 2.1 cm^2^, by placing 0.1 mm‐thick stainless steel inserts (Advanced Chemical Etching Ltd, Shropshire, UK) in specified locations on the membrane. These were placed using a custom‐built mould (Rapid Design & Fabrication Facility, UCL) with a vacuum pick‐up tool (624–9829, RS Components, Northants, UK) and held in place to fix location using a medical grade glue (Loctite 4011, Henkel AG & Co, KGaA, Berkeley, CA). The perspex chamber is equipped with three ports, one for the feed from a syringe pump (Harvard PHD Ultra Syringe pump 4400, Harvard Apparatus Ltd, Edenbridge, UK), a second for a pressure sensor (40PC100G2A, Honeywell Sensors and Control, Golden Valley, MN), which is connected to a multifunction data acquisition device (National Instruments Corporation Ltd, Berkshire, UK), and a third for a thermocouple data logger (EL‐GFX‐TC, Lascar Electronics Ltd, Wiltshire, UK).

Constant pressure operation was enabled using the pressure sensor to determine the syringe pump setting (LabVIEW 2015, National Instruments Corporation Ltd). All experiments were carried out at constant transmembrane pressure of 1.0 bar and at a controlled temperature of 20.0 ± 0.3°C by circulating cooling water (10°C) through the jacketed‐base of the USD device.

### 2.3 Experimental methods

For the pilot‐scale TFF experiments, 0.1 M NaOH was first drained from the system and the inline cassette was replaced by a new one. Two washing stages (initially full recirculation and then open permeate line) were performed using ~1.0 L of ultra‐pure water (0.22 µm filtered from a MilliQ station) to flush the cassette storage solution at a cross flow rate (*Q*_F_) of 0.4 L/min. The membrane resistance (R_M_) was determined from the flux rate (*Q*_D_) of ultra‐pure water required to reach Δ*P*_TMP_ of 1.0 bar (In all cases steady‐state conditions were achieved within 15 min and the use of the membranes was continued). The system was flushed with ~1.0 L of diafiltration buffer, 10 mM Tris Acetate (pH 5.4) at a cross *Q*_F_ of 0.4 L/min and then manually filled, avoiding foam formation, with a feed volume of 0.89 L of the antibody solution. Diafiltration operation was performed at a Δ*P*_TMP_ of 1.0 bar. The diafiltration buffer was fed to maintain feed volume constant for a total of seven diafiltration volumes (DV). Permeate flux (J) was determined by the measurement of the permeate fraction weights every 0.1 min for the *Q*_F_ tested. On completion the protein concentration in the retentate was recorded. Cleaning was performed by flushing 0.1 M NaOH for 30 min at *Q*_F_ = 0.4 L/min. The system was primed and stored at room temperature in 0.1 M NaOH.

For each USD run, a new filter disc was placed during the set‐up of the USD system. A wash stage was performed using 9 mL of ultra‐pure water for 30 min. The *R*_M_ was determined from the *Q*_D_ of ultra‐pure water required to reach Δ*P*_TMP_ of 1.0 bar. Where steady‐state conditions were not achieved within 15 min, the filter disc was discarded. The USD system was flushed with 9 mL of diafiltration buffer at a *Q*_D_ of 86 LMH (0.3 mL/min) before manually filling the stirred cell chamber with a feed volume of 1.7 mL of the antibody solution, i.e., its maximum capacity. This prevented the formation of air‐liquid interfaces and consequently the development of a vortex even at high disc speeds.

The *Q*_F_ of the diafiltration buffer was adjusted to result in a Δ*P*_TMP_ of 1.0 bar until completion of seven DV. Permeate flux (*J*) was determined from the diafiltration pump flow rate and recorded every 0.02 min for each disc speed tested. Upon completion of the USD test, the concentration of the antibody solution in the chamber was recorded and a visual inspection made for any significant protein deposits remaining. Cleaning was performed by flowing 0.1 M NaOH at a *Q*_D_ of 86 LMH (0.3 mL/min) at *N* = 3,000 rpm. The system was then drained and rinsed with water and the membrane was discarded.

### 2.4 Computational methods

Shear rate analysis for flow in the USD‐stirred cell was performed using computational fluid dynamics simulations (Ansys, Inc., CFX version 17.0, Canonsburg, PA, run on an Intel^®^ Xeon^®^ CPU E5–2687W with two processors of 3.4 GHz with 256 Gb RAM memory). Because of rotational symmetry, only a quarter of the disc was modelled as a 3D cross section. The rotating disc was set as an individual wall boundary with no slip. The model selected was shear stress transport, i.e., a combination of k‐ε, which provides an initial overview of the flow conditions and k‐ω, which improves accuracy of the results by solving the basic momentum transport equations in radial, axial, and azimuthal components, particularly near the chamber wall and the boundaries.

All simulations were meshed with 11 million elements and iterated until reaching the defined number of iterations of 200 and converging within a root mean square (RMS) of 1e^−4^ s^−1^ for the shear rate values. Each simulation required ~9 hr process time to complete. The chosen mesh element size and convergence conditions gave reasonably accurate results at an acceptable process time for each simulation.

A characteristic average shear rate was the chosen basis for scale translation between the USD and the pilot‐scale TFF systems. In the USD system this was defined as the average shear rate in a 0.1 mm height of fluid above the active area of the membrane surface. This height is similar in magnitude to an effective individual channel height in flat‐sheet cassettes (Rayat et al., 2014).

For the pilot‐scale TFF system, the shear rate was estimated using Equation (1), where the axial pressure drop, Δ*P*_axial_, was measured experimentally with ultra‐pure water and the cassette hold‐up volume (V) was obtained from the manufacturer (EMD Millipore) (Binabaji, Ma, Rao, & Zydney, 2015).

(1) $${\overset{®}{\text{y}}}_{\text{av}}\text{ = }\sqrt{\frac{{\text{Q}}_{\text{F}}\text{ ∙ }{\text{∆}\text{P}}_{\text{axial}}}{\text{V}\text{ ∙ }{\text{μ}}_{\text{F}}}}$$

## 3 RESULTS AND DISCUSSION

### 3.1 Computational fluid dynamics

**Figure 2:**
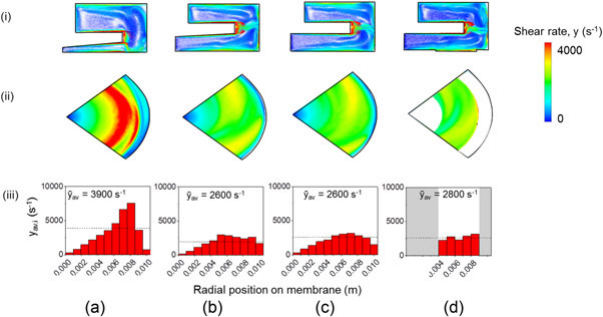
Effect of USD device design on shear rate profiles using CFD simulations. Different disc designs (angle *ϴ*) and heights above the membrane at the disc centre (*D*_1_) and edge (*D*_2_) are studied: (a) *D*_1_ = 0.6 mm, *D*_2_ = 1.1 mm and *ϴ* = 3.4°; (b) *D*_1_ = 1.6, *D*_2_ = 2.0 mm and *ϴ* = 3.4°; (c) *D*_1_ = *D*_2_ = 2.0 mm and *ϴ* = 0°; (d) *D*_1_ = *D*_2_ = 2.0 mm and *ϴ* = 0° with blanking of inner and outer sections of the membrane. Shear rate flow vectors are shown to be 0.1 mm above the membrane surface in two different planes: (i) cross‐sectional, (ii) longitudinal, and (iii) as the profile of mean shear rates of fluid elements for a disc speed of 2,500 rpm and viscosity of 0.0013 Pa s. A characteristic overall average shear rate (*ȳ*_av_) over the membrane is given for each design [Color figure can be viewed at wileyonlinelibrary.com]

The flow conditions were characterized for a range of disc designs and placements. These were varied with a view to achieving an approximately uniform shear rate across the membrane surface as is likely to be present during fluid flow in the pilot‐scale TFF membrane cassette. Some examples studied for the USD device operating in the mid‐speed range available are given in Figure 2. The initial USD design (Figure 2a) was as studied previously (Ma et al., 2009) with a conical disc close to the membrane surface with the assumption that a narrowing gap might partially offset the decreasing angular velocity to give a more uniform shear rate. However, the majority of radial flow is predicted to occur above the disc with just one flow vortex beneath the edge of the disc and the chamber wall (Figure 2a [i] and [ii]). A sharp shear rate profile results with *y*_min_ = 300 and *y*_max_ = 7,600 s^−1^ (Figure 2a [iii]).

Raising the disc (Figure 2b) allowed the flow vortex to cover greater portions of the membrane, i.e., rather than have the majority of radial flow above the disc (Figure 2b [i]). A critical minimum distance (*D*_2_) of at least 1.35 mm between membrane surface and disc edge was found for a range of disc angles to be necessary to allow radial flow over the membrane surface and improve the uniformity of shear rate albeit still with a sharp profile (*y*_min_ = 200 and *y*_max_ = 3,000 s^−1^, Figure 2b [iii]). Further simulations, for example, Figure 2c showed that the disc angle did not impact flow over the membrane provided the critical distance (*D*_2_) between the disc and membrane was maintained.

**Figure 3:**
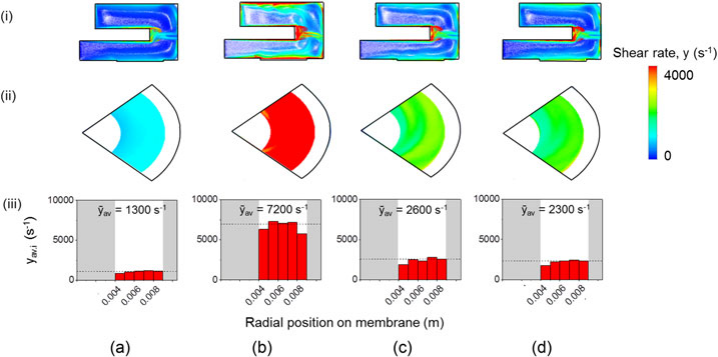
Effect of disc speed and viscosity on shear rate profiles for USD configuration shown in Figure 2d. The combination of disc speeds and viscosities studied are as follows: (a) 1,300 rpm and 0.0013 Pa s; (b) 5,000 rpm and 0.0013 Pa s; (c) 2,500 rpm and 0.0016 Pa s; and (d) 2,500 rpm and 0.0020 Pa s. Figure 2d shows profiles obtained at 2,500 rpm and 0.0013 Pa s. See Figure 2 legend for further description [Color figure can be viewed at wileyonlinelibrary.com]

For the design shown in Figure 2c, the regions were identified where the shear rate over the membrane was predicted to be less than 0.8 *y*_av,_ i.e., a central region and an outer annulus. These regions were blanked (Figure 2d) resulting in an acceptably uniform shear rate over the remaining exposed membrane surface with *ȳ*_av_ of 2,800 s^−1^ (*y*_min_ = 2,300 and *y*_max = _3,000 s^−1^, Figure 2 d [iii]). The use of 0.1mm‐thick blanking pieces led to little change in the flow patterns in the USD chamber and no significant regions of low shear adjacent to the membrane and at the stepped edge of the blanking pieces (Figure 2d [i]). The final design is shown in Figure 2d was studied for other disc speeds and viscosities with examples shown in Figure 3. Similarly uniform shear profiles over the membrane are observed in all cases.

### 3.2 Design of scale‐down TFF experiments

**Figure 4:**
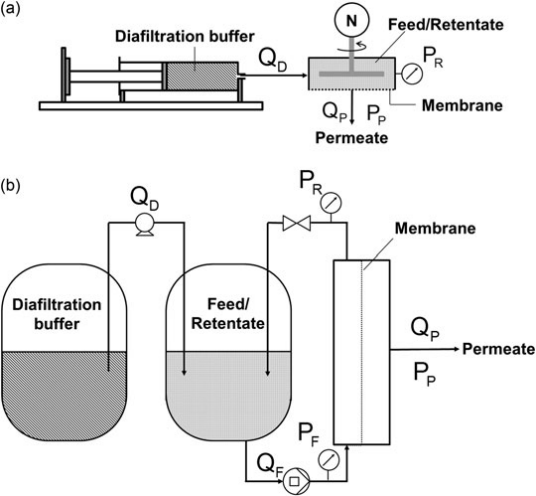
Schematic representation of (a) ultra scale‐down (USD) and (b) pilot‐scale TFF membrane systems (drawings are not to scale). In this study, the volume required for the USD system is approximately 520‐fold smaller than for the pilot‐scale setup. A comparison between these systems is given in Table 1

Comparison of USD and pilot‐scale TFF systems was performed at a constant volumetric membrane loading; 8.1 L of feed/m^2^. Table 1 further describes the properties of the two systems and these are represented schematically in Figure 4 for the resultant USD membrane area as defined in Figure 2d.

**Table 1:** Comparison of the USD and the pilot‐scale TFF systems and their operation

	Ultra scale‐down (USD) system	Pilot‐scale TFF system
Effective membrane area, m^2^	0.00021^a^	0.11^b^
Feed volume of material required per experiment, L	0.0017	0.89
Pressure drop characterisation	$\text{∆}{\text{P}}_{\text{TMP}}\text{ = }{\text{P}}_{\text{R}}-{\text{P}}_{\text{P}}$	$\text{∆}{\bar{\text{P}}}_{\text{TMP}}\text{ = }\frac{\left(P_{F}+P_{R}\right)}{\text{2}}-{\text{P}}_{\text{P}}$
Operation	Constant Δ*P*_TMP_ ^c^	Constant Δ*P*_TMP_ ^d^
Shear rate characterisation	*f* (*N*) using CFD	*f* (Q_F_, Δ*P*_axial_)

^a^ See Section 2 for further details.

^b^ Manufacturer’s definition.

^c^ Achieved using feedback loop control to adjust syringe pump flux rate.

^d^ Achieved using adjustable pinch‐valve on retentate outlet stream.

**Figure 5:**
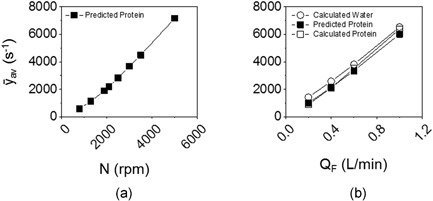
Comparison of shear rate (*ȳ*_av_) relationships for (a) USD as a function of disc speed using CFD analysis and (b) pilot‐scale TFF using measured ∆*P*_axial_ for set *Q*_F_ values. In (a), *ȳ*_av_ is predicted using CFD for the mAb solution to be studied (*µ* = 0.0013 Pa s). In (b), calculated *ȳ*_av_ for water (○) is obtained from the measured ∆*P*_axial_ of water and *µ* ( = 0.0010 Pa s). The predicted *ȳ*_av_ for the protein solution (■), is obtained from the measured ∆*P*_axial_ of water, assuming ∆*P*_axial_ ≠ *f*(*µ*) for transitional and turbulent flow (1,400 < Re < 7,000), and *µ* = 0.0013 Pa s, using Equation (1). The calculated *ȳ*_av_ for the protein solution (□) is determined from the measured ∆*P*_axial_ of protein and *µ* = 0.0013 Pa s

Figure 5a summarises the predicted average shear rate versus disc speed for the USD device design chosen for further study (Figure 2d) for the process stream studied here (*µ* = 0.0013 Pa s). The full CFD results may be correlated to give ȳ_av_ ∝ *N* ^1.37^ *µ* ^−0.46^ (for 1,300 < *N* < 5,000 rpm, 0.0013 < *µ* < 0.0020 Pa s) similar to a relationship which may be expected for turbulent flow (e.g., mean velocity gradient ∝ (*ε*/*µ*)^0.5^ ∝ *N* ^1.5^ *µ* ^−0.5^, where ε is power dissipated per unit volume and power dissipated ∝ *N* ^3^ for stirred vessels). A previous study by Ma et al. (2009) similarly correlated *ȳ*_av_ ∝ *N* ^1.5^ for a USD device with design as shown in Figure 2a. The strategy for the shear rate characterisation in the pilot scale TFF system is based on pressure drop versus flow rate characteristics using water. This relationship is used with the mAb solution viscosity to predict the shear rates for the mAb solution (Equation (1) and Figure 5 legend). These agree with the calculated shear rates using the mAb solution in the pilot‐scale TFF system. Different strategies will be needed when dealing with more viscous protein solutions where the flow will be in the laminar region unlike for these studies (Binabaji, Ma, Rao, & Zydney, 2016). The predicted shear rates for the mAb solution in the pilot‐scale TFF system (Figure 5b) are within the range of those predicted for the USD device (Figure 5a).

**Figure 6:**
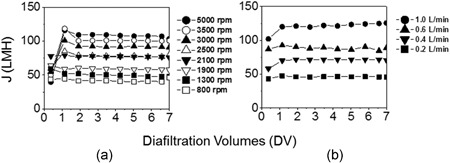
Flux versus diafiltration volume profiles for (a) USD device operating at different disc speeds (see inset) and (b) pilot‐scale TFF at different cross flow rates (see inset). Both systems operated at the same Δ*P*_TMP_ of 1.0 bar. Fresh membranes are used for each run. Data for single runs are reported here obtained from a moving average of raw data (*m* = 100), where SD is ~1%. The protein solution used is a 12 g/L mAb‐1 solution prepared in 10 mM Tris Acetate pH 5.4. The diafiltration buffer was 10 mM Tris Acetate pH 5.4

A diafiltration operation was used to compare the flux performance of both the USD and pilot‐scale TFF systems for varying disc speeds or cross flow rates, respectively (Figure 6). Similar profiles were achieved with higher steady state flux being achieved for increased flow conditions as expected. In both cases the membranes behaved similarly with comparable flux profiles as a function of increasing diafiltration volumes. For both TFF and USD a decline in protein concentration in solution was observed on completion of diafiltration, this varied from 10% to 33% for USD and 6% to 19% for TFF. No visible loss protein deposits were observable in the USD device and little additional protein was recovered by water flushing.

### 3.3 Using gel resistance as a comparative measure

**Figure 7:**
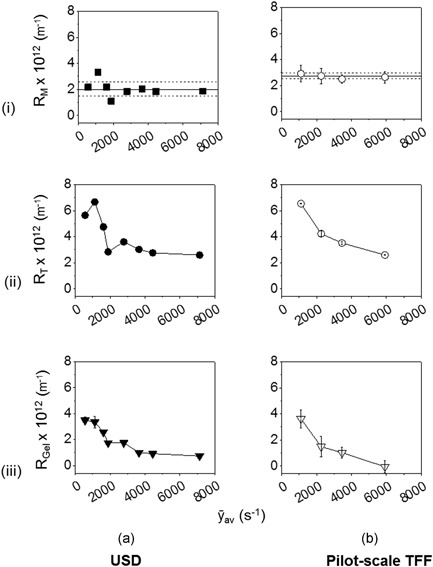
Effect of (i) membrane resistance (R_M_); (ii) steady‐state total resistance (*R*_T_) derived from Figure 6 using Equation (2); and (iii) steady‐state gel resistance (*R*_Gel_) using Equation (3) as a function of flow conditions represented by average shear rate, ȳ_av_, related to USD disc speed (Figure 5a), to pilot‐scale TFF cross flow rate (see Figure 5b). See Figure 6 legend for the protein solution and buffers used. The viscosity of permeate (µ_P_) in Equation (2) was assumed to be the same as for the diafiltration buffer. Resistance values are for USD (*n* = 1) and pilot‐scale TFF experiments (*n* = 1). The range bars are for the s.d. values of the resistance measurements in the stable region (3.5 < DV < 7.0). (i) gives mean (^**_ _ _**^) ± 1 SD (‐ ‐) for all membranes used. Note vertical y axes runs to − 0.6 × 10^12^ m^−1^ to aid visualization

Experiments on both scales were compared using gel resistance, an engineering parameter which, after adjusting for membrane variability, is based on transmembrane pressure, flux and material viscosity measurements. The rheology of the mAb‐1 solutions tested showed Newtonian behaviour across the studied range of viscometer shear rates between 1,000 and 7,000 s^−1^ (data not shown here). Both clean membrane resistance (*R*_M_) and total resistance (*R*_T_) were calculated from steady‐state flux measurements (Equation (2)). The greater range of the USD disc membranes can be seen in Figure 7i (i.e., ±1.0 × 10^12^ m^−1^ compared with ±0.3 × 10^12^ m^−1^).

(2) $$R_{x}=\frac{{\Delta \bar{P}}_{TMP}}{J_{SS}\cdot \mu_{P}}$$

(3) $${\text{R}}_{\text{Gel}}\text{ = }{\text{R}}_{\text{T}}-{\text{R}}_{\text{M}}-{\text{R}}_{\text{F}}$$

The resultant gel resistance (*R*_Gel_) is given by Equation (3) assuming *R*_F_ = 0. The values of *R*_M_, *R*_T_ and *R*_Gel_ for various shear rates are shown in Figure 7. Similar effects were observed of change in shear rate on gel resistance in the USD and the pilot‐scale TFF systems (Figure 7iii). At lower shear rates, protein molecules flow towards the membrane surface and are less effectively swept away due to weaker cleaning action contributing to the formation of the gel layer. Gel resistance dominates the total resistance distribution at low shear rates (<2000 s^−1^). At higher shear rates, membrane resistance dominates.

(4) $$\delta =\frac{{\bar{R}}_{M}-R_{M}}{R_{T}}$$

(5) $$\hat{\text{J}}\text{ = }{\text{J}}_{\text{SS}}\text{(1}-\text{δ)}$$

**Figure 8:**
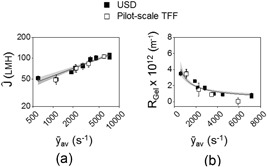
Comparison of USD and pilot‐scale TFF performance using CFD predicted *ȳ*_av_ for USD to match with experimental *ȳ*_av_ for pilot‐scale TFF: (a) normalised steady‐state flux rates, Ĵ and (b) gel resistance, *R*_Gel_. Normalised steady‐state flux (Ĵ) is given by Equation (5) using steady‐state flux (*J*_SS_) in the stable region (3.5 < DV < 7.0) from Figure 6 (factor 1−δ varies for USD from 0.8 to 1.3 and for TFF from 0.9 to 1.1). Gel resistance (*R*_Gel_) is obtained from Figure 7iii. Correlations obtained are: (a) Ĵ ∝ *ȳ*_av_^0.36^, *R* ^2 = ^0.89; and (b) *R*_Gel_ ∝ ȳ_av_^0.63^, *R* ^2 = ^0.81. Range bars are obtained from s.d. of values in the stable region (3.5 < DV < 7.0). Note vertical *y* axis in (b) runs to − 0.6 × 10^12^ m^−1^ to aid visualization. See Figure 6 legend for details of the protein solution and buffers used

An adjustment factor, δ (Equation (4)) was determined for each experiment to account for membrane variability. Normalised steady‐state flux rates (Ĵ) Equation (5) and the gel resistance (*R*_Gel_) were used to compare experimental runs of both systems at equivalent characteristic average shear rates, *ȳ*_av._ There is good agreement in Ĵ and *R*_Gel_ data as shown in Figure 8a,b between USD and pilot‐scale TFF experimental runs. The coefficient of 0.36 ± 0.04 relating flux and shear rate (Figure 8a) is within the published range of 0.33–1.33 for different protein ultrafiltration applications (Cheryan, 1998).

## 4 CONCLUSION

The limited availability of material for process development studies, particularly with high concentration antibody solutions, poses a challenge to identify optimum operating conditions for a successful scale‐up. A novel scale‐down approach has been presented in this study to predict diafiltration performance of a typical pilot‐scale TFF system using flat‐sheet membrane cassettes by implementing USD technologies. CFD simulations have been used to characterize flow patterns of the chamber in the USD system in terms of average shear rate. The match between the USD and the pilot‐scale TFF system was done using a characteristic average shear rate as the basis for scale translation. Good agreement of data was observed when comparing gel resistance and flux of equivalent experimental runs between scales. This article describes a proof‐of‐concept study of how USD may be used to determine the effect of operating variables on membrane performance and hence enhance the effectiveness of subsequent pilot‐scale experimentation. However the lack of comparability of protein loss between the USD and TFF devices is possibly due to the increased surface area to volume in the USD device. If the USD device is also to be used in preparative mode, there is a need for redesign to give equivalence to the TFF system.

**Figure 9:**
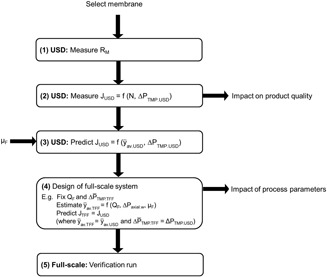
Suggested use of the USD system to gain early insight into processing of a new candidate and to help determine the design of a full‐scale TFF system. Step 2 may include a study to establish a design space, for example, *N* (800–5000 rpm) and ∆*P*_TMP.USD_ (0.4–1.6 bar) and also to give an indication of impact on product quality (to be reported in future paper). Step 3 for USD *ȳ*_av.USD =_ *f* (*N* ^1.37^ *µ*_F_^−0.46^). In Step 4 the predicted impact of ∆*P*_TMP.TFF_ and *Q*_F_ on the performance may also determine their relevance as critical process parameters

Figure 9 summarises how the USD system may be used to help contribute to the design of full‐scale TFF operations. USD experiments may be conducted to determine membrane performance using flux rate even when limited amounts of process material are available. The resultant engineering correlations may be used to predict full‐scale TFF operation for that process material provided design features of the full‐scale system are known, for example, operating characteristics with water. Membrane performance other than flux rate might include the transmission of contaminants determining the extent of diafiltration needed. To date the only measure used to validate the shear rate estimations has been the flux rate and the gel resistance. Other criteria may be: product recovery, product quality attributes affected by shear stress, and concentration operations using higher concentrations, which will be the subject of a future study.

**NOMENCLATURE**
*D*_1_: distance between the centre of the disc and the membrane surface
*D*_2_: distance between the edge of the disc and the membrane surface
*J*: permeate flux rate
*N*: disc speed
*P*: fluid pressure
Δ*P*: pressure differential across membrane
*Q*: flow rate
*R*: resistance
*V*: cassette hold‐up volume
*Ø*: diameter
*ϴ*: disc angle
*δ*: adjustment factor
*µ*: viscosity
*ȳ*: characteristic shear rate

**SUBSCRIPTS**
av: average
D: diafiltration buffer
F: feed
Gel: gel
M: membrane
P: permeate
R: retentate
SS: steady‐state
T: total
TMP: transmembrane pressure
W: water
